# Dynamics of a New Strain of the H1N1 Influenza A Virus Incorporating the Effects of Repetitive Contacts

**DOI:** 10.1155/2014/487974

**Published:** 2014-03-13

**Authors:** Puntani Pongsumpun, I-Ming Tang

**Affiliations:** ^1^Department of Mathematics, Faculty of Science, King Mongkut's Institute of Technology Ladkrabang, Chalongkrung Road, Ladkrabang, Bangkok 10520, Thailand; ^2^Department of Mathematics, Faculty of Science, Silpakorn University, Nakhon Pathom 73000, Thailand; ^3^Department of Physics, Faculty of Science, Mahidol University, Bangkok 10400, Thailand

## Abstract

The respiratory disease caused by the Influenza A Virus is occurring worldwide. The transmission for new strain of the H1N1 Influenza A virus is studied by formulating a SEIQR (susceptible, exposed, infected, quarantine, and recovered) model to describe its spread. In the present model, we have assumed that a fraction of the infected population will die from the disease. This changes the mathematical equations governing the transmission. The effect of repetitive contact is also included in the model. Analysis of the model by using standard dynamical modeling method is given. Conditions for the stability of equilibrium state are given. Numerical solutions are presented for different values of parameters. It is found that increasing the amount of repetitive contacts leads to a decrease in the peak numbers of exposed and infectious humans. A stability analysis shows that the solutions are robust.

## 1. Introduction

Influenza virus can be divided into three types: influenza A, B, and C. The virus is spread when droplets from a cough or sneeze of an infected person is propelled through the air and deposited on the mouth, nose, or eye of persons nearby. The virus can also land on surfaces in the surrounding environment and survive on the surfaces for 24 hours if the surface is hard and around 20 minutes if the surface is soft. The subtype H1N1 (2009) influenza virus is one of the influenza viruses that can cause respiratory illnesses. It is related to the virus which caused the 1918 flu pandemic. It is now called the swine flu virus because the virus causing pigs in the United States to become sick shortly after the 1918 pandemic was identified as being the H1N1 virus [1].

Between 1997 and 2002, new strains of the H1N1 (three different subtypes and five different genotypes) started to emerge as the causes of influenza among pigs in North America. By 2009, a new virus began to spread among people in the American continent. It was identified as being related to the virus infecting the pigs in Mexico and was labeled as the H1N1 (2009) virus (WHO Bulletin, 24 April 2009). This virus quickly spread to 160 countries and territories. By mid-2009, there were 135,000 cases and 816 deaths. The H1N1 (2009) virus has spread from the American continent to the rest of the world, that is, Europe, the Middle East, Asia, the Pacific and Africa, making this disease a pandemic.

In Thailand, H1N1 (2009) was isolated from pigs with an influenza-like symptom in 1990 [2]. In 2005, a new subtype of the virus H1N1 was isolated from pigs in Saraburi province [3]. Thai people quickly became sick with this virus. The most common clinical symptoms of the 2009 H1N1 influenza A pandemic were fever, cough, sore throat, malaise, headache, vomiting, and diarrhea. Other common symptoms are chills, myalgias, and arthralgias [4, 5]. These are also common to the symptoms of seasonal fever, so that proper diagnoses require laboratory tests. The most active areas of pandemic influenza virus transmission are now in parts of Southeast Asia, West Africa, and in the tropical zone of the Americas. The Centers for Disease Control and Prevention (CDC) recommends the antiviral drugs Tamiflu (oseltamivir) or Relenza (zanamivir) for treatment and prevention of infection with the swine flu virus. Antiviral drugs work best if started within 2 days of symptoms [6].

New strains of the influenza A virus constantly appear. For example, on March 29, 2013, the Chinese Center for Disease Control and Prevention discovered three human infections with an avian influenza A (H7N9) [7]. A total of 131 confirmed cases of human infection with avian influenza A (H7N9) virus have been reported to the WHO by China National Health and Family Planning Commission and one case by the Taipei Centers for Disease Control (Taipei CDC). Although cases have been reported in both sexes and across a wide range of ages, most cases have occurred among middle-aged and older men. Thirty-two people have died, and most of the other cases are considered severe. In addition to the case reported by Taipei CDC (with a history of recent travel from Jiangsu), cases have been reported from Anhui, Fujian, Henan, Hunan, Jiangsu, Jiangxi, Shandong, and Zhejiang and the municipalities of Beijing and Shanghai. Seasonal influenza A virus continues to spread among persons in area where H7N9 cases have been detected. The Chinese Centers for Disease Control and Prevention had reported that rates of influenza-like illness are consistent with expected seasonal flu levels.

Biological factors such as the duration of infectious period and social factors can influence the spread of this disease. Repetitiveness of contacts is also known to be the relevant factors effect to the transmission of droplet or contact transmitted diseases. In 2009, Smieszek et al. showed that random mixing models provide acceptable estimates of the total outbreak size if the number of contacts per day is high [8]. Klinkenberg et al. [9] presented the strategy for emergency vaccination during an epidemic of classical swine fever virus (CSFV) and formulated a mathematical model of CSFV transmission between pig herds which quantifies the effect of control strategies with and without vaccination and estimate the model parameters from data of the 1997/1998 CSFV epidemic in The Netherlands.

Nishiura [10] showed that predictions based on mathematical modeling have two components: projections and forecasting. Projections involve the simulation of what would happen if certain assumptions and hypotheses are made while a forecasting is a quantitative prediction of what will happen in the future. Weather forecasting is an attempt to predict what the weather at some times in the future will be. This can be done if the future is several days. Long term forecasting is impossible since the equations used for the forecasting are input sensitive equations meaning that the solutions depend on values of the initial values of the input which depend on random events. Prior to the 2009 pandemic, mathematical modeling offered projections on “what if” scenarios. Keeping this in mind, Chowell et al., [11] used mathematical modeling to make projections on what would happen if nonmedical interventions such as school closing were used to control the spread of 2009 influenza A/H1N1 pandemic in Mexico. Zhou and Guo [12] made projections on the spread of A/H1N1 virus when a vaccination program was used to control the pandemic. Jin et al., [13] carried an analysis of an A/H1N1 epidemic if it occurred on a network and what it would be if different vaccination policies were applied at different points on the network. Prosper et al., [14] looked at the control strategies for the pandemic H1N1 influenza in a background of seasonal influenza which affects a greater number of people.

In this paper, we study the transmission of a new H1N1 influenza A virus in human with the effect of repetitive contact taken into consideration. A statistical model was developed to estimate the social contact network within a high school using friendship network data and a survey of contact behavior [15]. We consider the transmission of a new strain of the H1N1 influenza A virus with the effect of repetitive contact included by constructing the mathematical model. We employ an SEIQR model (*S* denoted susceptible human; *E*, exposed; *I*, infectious; *Q*, quarantine, and *R* denotes a recovered human) [16] to describe the transmission of the new virus in the population. Any new strains of influenza pose a danger to society since the populace does not have any immunity to the virus and therefore there is a greater chance that some of the infected people will die. We have taken into account the possibility that some of the infected populace will die in the model. We have simulated the effects of varying the number of repetitive contacts, the percentage of infected individuals being put in quarantine and allowing for infected individuals to die from the disease.

## 2. Dynamical Equations

We consider the transmission of H1N1 influenza A virus' in human. We have included the effect of repetitive contact and taken into account that people having a prior illness have a higher mortality rate. We begin by defining

 
*S*(*t*) as being the number of susceptible persons at time *t*;
 
*E*(*t*) as the number of exposed persons at time *t*;
 
*I*(*t*) as the number of infectious persons at time *t*;
 
*Q*(*t*) as being the number of quarantine persons at time *t*;
 
*R*(*t*) as being the number of recovered persons at time *t*.

Our model is based on the standard SEIQR (susceptible, exposed, infected, quarantine, and recovered) model for contact transmission. A susceptible human becomes an exposed person if he comes in contact, direct or indirect (i.e., by touching a surface on which the new strain of the H1N1 virus may lie). If *C* is the number of contacts a human makes with someone who could pass on the virus, then *C*(*E*(*t*) + *I*(*t*)) is the number of contacts which could result in a person being exposed to the virus. *S*(*t*)/*N*(*t*) (*N*(*t*) being the total number of humans at time *t*) is the probability that the person being contacted is a susceptible human. If a certain percentage of the contacts are repeated ones, then the increase in the number of exposed human is given by

(1) $$\begin{array}{c} \left(1-r_{c}\right)CS\left(t\right)\frac{\left(E\left(t\right)+I\left(t\right)\right)}{N\left(t\right)}, \end{array}$$

where *r*_*c*_ is the percentage of contacts which are repetitious. These contacts do not lead to a new exposed human. It should also be noted that just because a person comes in contact with an exposed person, he may not come in contact with the virus. We also do not know at what point a person exposed to a virus becomes infectious, we must assume that an exposed person could transmit the virus before he is placed in the infectious class.

The change in the number of humans in any group is equal to the number entering the group minus the number leaving the group. Thus the change in the number of susceptibles is equal to the number of people entering into the population (*λ*_*h*_ is the birth rate of humans) minus the number of susceptibles being exposed to people (given by (1)) and the number of susceptibles dying from natural causes.

Consider

(2) $$\begin{array}{c} \frac{d}{dt}S\left(t\right)=\lambda_{h}N\left(t\right)-\left(1-r_{c}\right)CS\left(t\right)\frac{E\left(t\right)+I\left(t\right)}{N\left(t\right)}-d_{n}S\left(t\right). \end{array}$$

The number of humans exposed to the N1H1 virus is equal to the number of susceptibles exposed (again given by (1)) to the virus minus those who recover, who become infectious, and who die (by a natural cause or an induced cause); that is,

(3) ddtE(t)=(1−rc)CS(t)(E(t)+I(t))N −(ρ+1IP+dn+δdh)E(t).

In this equation, *δd*_*h*_ is the additional death rate caused by the new virus for which the human population has no immunity. The change in the number of infectious humans is equal to the percentage of the exposed humans, who develop into infectious humans minus the number of infectious humans who are placed in quarantine, who die from any cause and who recover,

(4) $$\begin{array}{c} \frac{d}{dt}I\left(t\right)=\frac{1}{\text{IP}}E\left(t\right)-\left(\alpha +\gamma +d_{n}+\delta d_{h}\right)I\left(t\right). \end{array}$$

The change in the number of humans who are quarantine is equal to the percentage of infectious humans, who are placed into quarantine minus the number who die or recover,

(5) $$\begin{array}{c} \frac{d}{dt}Q\left(t\right)=\gamma I\left(t\right)-\left(k+d_{n}\right)Q\left(t\right). \end{array}$$

Finally, the change in the number of recovered humans is equal to the number of exposed humans, infectious humans, and quarantine humans recover minus the number of recovered humans who die from natural causes,

(6) $$\begin{array}{c} \frac{d}{dt}R\left(t\right)=\rho E\left(t\right)+\alpha I\left(t\right)+kQ\left(t\right)-d_{n}R\left(t\right). \end{array}$$

The total number of humans is the sum of the five population groups at time *t*; that is,

(7) $$\begin{array}{c} N\left(t\right)=S\left(t\right)+E\left(t\right)+I\left(t\right)+Q\left(t\right)+R\left(t\right). \end{array}$$

Then (7) becomes

(8) $$\begin{array}{c} \frac{d}{dt}N\left(t\right)=\lambda_{h}N\left(t\right)-d_{n}N\left(t\right)-\delta d_{h}\left(E\left(t\right)+I\left(t\right)\right). \end{array}$$

With the assumption that *λ*_*h*_ = *d*_*n*_, then we have

(9) $$\begin{array}{c} \frac{d}{dt}N\left(t\right)=-\delta d_{h}\left(E\left(t\right)+I\left(t\right)\right). \end{array}$$

**Figure 1:**
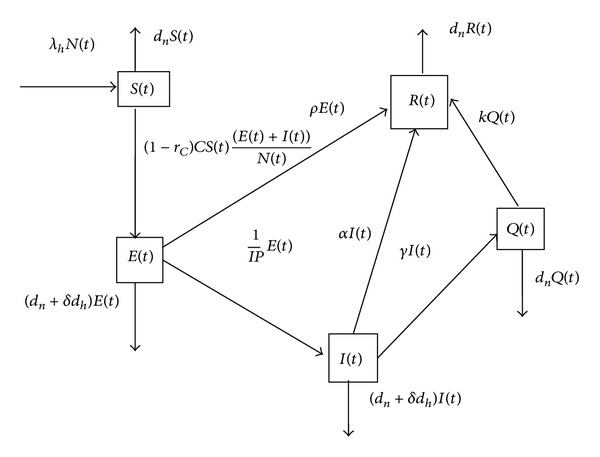
Flow chart of the dynamics in the model.

If the summation of *I*_*h*_ and *E*_*h*_ are not equal to zero, the human population will decline. The flow chart of this model is shown in Figure 1. A summary of the definitions of the parameters of our dynamical equations are given in Table 1. We reduce our dynamical equations by introducing the new variables:

(10) $$\begin{array}{c} S\left(t\right)=\frac{S\left(t\right)}{N\left(t\right)},\;\;e\left(t\right)=\frac{E\left(t\right)}{N\left(t\right)},\;\;i\left(t\right)=\frac{I\left(t\right)}{N\left(t\right)},\\ q\left(t\right)=\frac{Q\left(t\right)}{N\left(t\right)},\;\;r\left(t\right)=\frac{R\left(t\right)}{N\left(t\right)}. \end{array}$$

Taking the time derivative of a normalized population *x*(*t*) = *X*(*t*)/*N*(*t*), then

(11) $$\begin{array}{c} \frac{d}{dt}x\left(t\right)=\frac{d}{dt}\frac{X\left(t\right)}{N\left(t\right)}=\frac{1}{N\left(t\right)}\frac{d}{dt}X\left(t\right)-x\left(t\right)\frac{1}{N\left(t\right)}\frac{d}{dt}N\left(t\right). \end{array}$$

Substituting (*d*/*dt*)*N*(*t*) = −*δd*_*h*_(*E*(*t*) + *I*(*t*)) in (11), then we obtain

(12) ddtx(t)=1N(t)ddtX(t)−x(t)1N(t)(−δdh(E(t)+I(t)))=1N(t)ddtX(t) −x(t)1N(t)(−δdh(e(t)N(t)+i(t)N(t)))=1N(t)ddtX(t)−x(t)(−δdh(e(t)+i(t))),ddtx(t)=1N(t)ddtX(t)+x(t)(δdh(e(t)+i(t))).

Therefore, we get

(13) $$\begin{array}{c} \frac{d}{dt}x\left(t\right)=\frac{1}{N\left(t\right)}\frac{d}{dt}X\left(t\right)+\delta d_{h}x\left(t\right)\left(e\left(t\right)+i\left(t\right)\right). \end{array}$$

With the above equation, the dynamical equations of the normalized populations are given by

(14) ddts(t)=λh−(1−rc)Cs(t)(e(t)+i(t)) −dns(t)+δdhs(t)(e(t)+i(t)),

(15) ddte(t)=(1−rc)Cs(t)(e(t)+i(t)) −(ρ+1IP+dn+δdh)e(t) +δdhe(t)(e(t)+i(t)),

(16) ddti(t)=1IPe(t)−(α+γ+dn+δdh)i(t) +δdhi(t)(e(t)+i(t)),

(17) $$\begin{array}{c} \frac{d}{dt}q\left(t\right)=\gamma i\left(t\right)-\left(k+d_{n}\right)q\left(t\right)+\delta d_{h}q\left(t\right)\left(e\left(t\right)+i\left(t\right)\right), \end{array}$$

(18) ddtr(t)=ρe(t)+αi(t)+kq(t) −dnr(t)+δdhr(t)(e(t)+i(t)).

## 3. Analytical Solutions

The steady state of our dynamical equations is given as follows:

(i) the disease free steady state *E*_0_ = (1,0, 0,0, 0),
(ii) the endemic steady state *E*_1_ = (s*, e*, i*, q*, r*), where
  (19) s∗=(dn(δdhi∗+(1IP))) ×(dn(1IP)−i∗(δ2dh2+α(δdh−C(1−rc))             +δdh(γ+(1IP)−C(1−rc))           −(dn+γ+(1IP))C(1−rc)))−1,e∗=i∗(α+dn+γ+δdh(1−i∗))δdhi∗+(1/IP),q∗=γi∗(δdhi∗+(1/IP))δdhi∗(α+δdh+γ+k)+(dn+k−δdhih∗)(1/IP),r∗=i∗(δdhi∗+(1/IP))dn(1/IP)−δdhi∗(α+δdh+γ+(1/IP)) ×(α−γk(δdhi∗+(1/IP))δdfi∗(α+δdh+γ−k)−(dn−δdh+k)(1/IP)   +(α+dn+γ+δdh(1−i∗))ρδdhi∗+(1/IP)),

where i* is the positive solution of the following equation:

(20) (δdhi∗(α+dn+γ+δdh(1−i∗)) ×(α+δdh+dn+γ+(1IP)))×(δdhi∗+(1IP))−1 −(α+dn+γ+δdh(1−i∗))(δdh+dn+(1IP)+ρ) +(dn(α+δdh+dn+γ+(1IP))   ×(δdhi∗+(1IP))C(1−rc)) ×(dn(1IP)−i∗(δ2dh2+α(δdh−C(1−rc))           +δdh(γ+(1IP)−C(1−rc))           −(dn+γ+(1IP))C(1−rc)))−1.

The local stability of each steady state is determined by the signs of all eigenvalues. The eigenvalues (*λ*) are solutions of the characteristic equation:

(21) $$\begin{array}{c} \left|J_{E}-\lambda I\right|=0, \end{array}$$

where *J*_*E*_ is the Jacobian matrix at the steady state and *I* is the identity matrix. If all eigenvalues produce the negative real parts, then the steady state is local stability.

### 3.1. For the Disease Free Steady State *E*_0_

The characteristic equation is given by

(22) $$\begin{array}{c} {\left(\lambda +d_{n}\right)}^{2}\left(\lambda +d_{n}+k\right)\left(\lambda^{2}+s_{1}\lambda +s_{0}\right)=0, \end{array}$$

where

(23) s1=α+2(δdh+dn)+γ+(1IP)+ρ−C(1−rc),s0=δ2dh2+γ(1IP)+(dn+γ)ρ +(dn+γ+(1IP))(dn−C(1−rc)) +α(δdh+dn+(1IP)+ρ−C(1−rc)) +δdh(2dn+γ+(1IP)+ρ−C(1−rc)).

The eigenvalues then become

(24) $$\begin{array}{c} \lambda_{1,2}=-d_{n},\;\;\lambda_{3}=-d_{n}-k,\\ \lambda_{4,5}=\frac{1}{2}\left(-s_{1}\pm \sqrt{s_{1}^{2}-4s_{0}}\right). \end{array}$$

From calculation, all eigenvalues have negative real parts for *R*_0_ < 1, where

(25) R0=(C(dn+γ+α(1IP)C(1−rc)+δdh(1−rc))) ×((α+δdh+dn+γ)(δdh+dn+(1IP)+ρ)    +(dn+γ+(1IP))Crc)−1.

### 3.2. For the Endemic Steady State *E*_1_

The characteristic equation is given by

(26) (λ+dn−δdh(e∗+i∗)(λ+dn−δdh(e∗+i∗)+k) ×(−(δdhi∗+(1IP))   ×(δ2dh2e∗(e∗+i∗)     −δdhe∗(d  n  +λ+(e∗+i∗)C(1−rc))     −(dn+λ)C(1−rc)s∗)) +(−α−δdh−dn+δdhe∗−γ−λ+2δdfi∗) ×((e∗+i∗)C(1−rc)   ×(−δdh+C(1−rc)s∗      +(λ+dn+(e∗+i∗)(C(1−rc)−δdh))      ×(λ+dn−δdh(2e∗+i∗−1)        +(1IP)+ρ−C(1−rc)s∗))))=0,

where s*, e*, i*, q*, and r* are defined in (19)-(20).

From evaluation, all eigenvalues have negative real parts for *R*_0_ > 1, where

(27) R0=(C(dn+γ+α(1IP)C(1−rc)+δdh(1−rc))) ×((α+δdh+dn+γ)(δdh+dn+(1IP)+ρ)    +(dn+γ+(1IP))Crc)−1.

${\bar{R}}_{0}=\sqrt{R_{0}}$ is defined as the basic reproductive number of the disease because it represents the average number of secondary cases that one primary case can produce [11].

## 4. Numerical Solutions and Discussion

In this paper, we are studying the effects of various effects of changing various on the transmission of the H1N1 influenza A virus. The factors we are interested in are the degree of repetitious, the increased death of the humans when infected by the virus, and the degree of quarantine of the infectious humans. Wu et al., [17] has estimated that the increase in the mortality rate due to the influenza virus during the pandemic was 11.1 per 100,000 each year. Most of the deaths occurred in people over 65 years old. Ejima et al., [18] has cautioned against the use of data taken in the early stages of an outbreak of a novel strain of influenza to estimate the case fatality ratio of the disease. Nishiura et al. [19] showed that the early prediction of the transmission potential of the pandemic (2009 H1N1) virus was wrong because of the sample size. One should not use a small sample to arrive at values for the parameters of the model. This has not stopped Nishiura et al. [20] from making predictions about the latest scare concerning the novel A (H7N9) virus which just appeared in China in 2013. Based on 20 confirmed cases, they came up with basic production number of 0.28, meaning that this disease would not become epidemic.

We are interested in the endemic steady state of our dynamical equations. The initial values of parameters used in this study are

(28) $$\begin{array}{c} d_{n}=\frac{1}{\left(365*65\right)},\;\;r_{c}=10\%,\;\;C=20,\\ \delta =3,\;\;d_{h}=\frac{1}{21},\;\;\text{IP}=5,\;\;\rho =\frac{1}{10},\\ \alpha =\frac{1}{14},\;\;\gamma =\frac{1}{7},\;\;k=\frac{1}{7},\;\;{\bar{R}}_{0}=3.53. \end{array}$$

**Figure 2:**
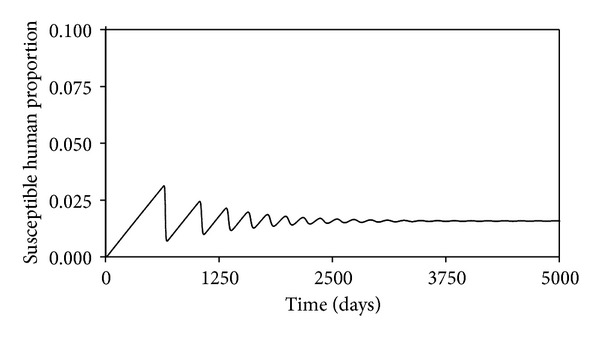
Time evolution of the susceptible human population.

**Figure 3:**
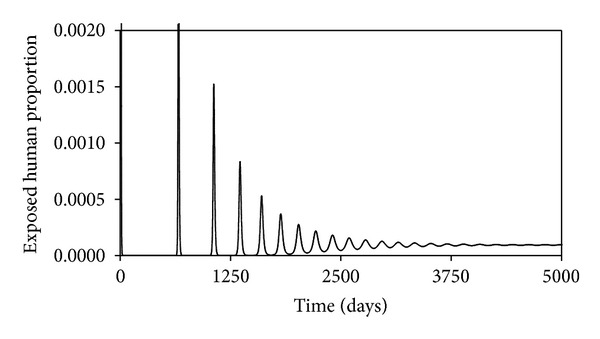
Time evolution of the exposed human population.

**Figure 4:**
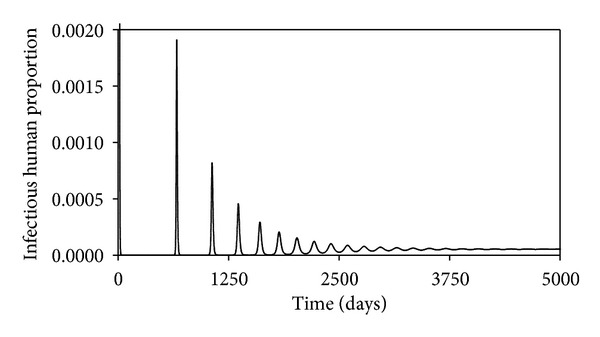
Time evolution of the infectious human population.

**Figure 5:**
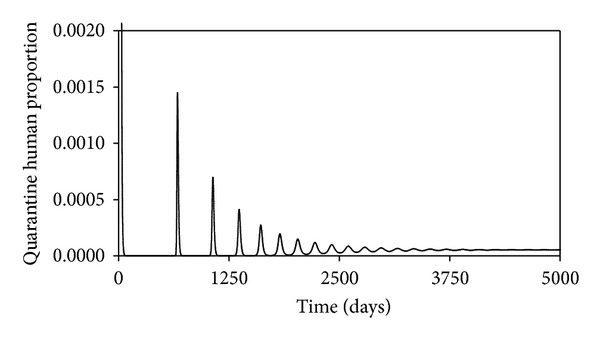
Time evolutions of the quarantine human population.

**Figure 6:**
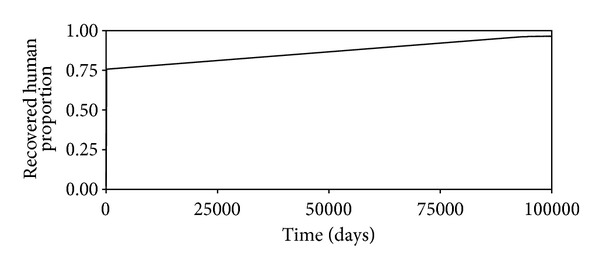
Time evolutions of the recovered human population.

These may not be the true values, but since we are interested in the changes in the projections of the behaviors of the different time evolutions of the different populations when the values of the parameters are changes, they will do. The time evolution of the susceptible human population is shown in Figure 2. We see that this decreases as time passes, reaching an equilibrium value of 0.016, which is what is predicted by (14). Figures 3, 4, 5, and 6 show the time evolutions of the exposed, infectious, quarantine, and recovered populations. We can see that the solutions oscillate to the endemic steady state. As we see, the equilibrium values of the four populations are 0.0000935, 0.000054, 0.000053, and 0.964; the values predicted by (15), (16), (17), and (18).

**Figure 7:**
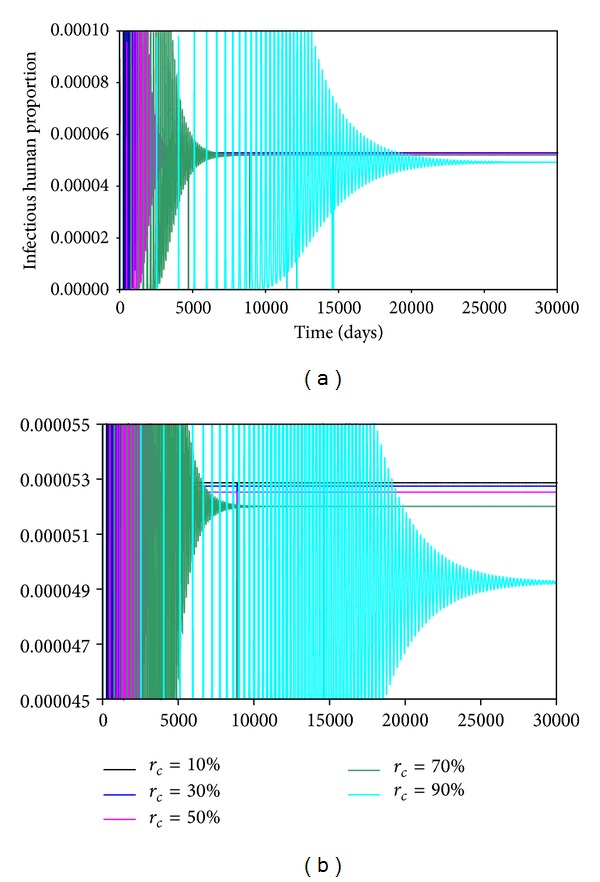
Time evolutions of the infectious human population for the different percentages of contact repetitions.

**Figure 8:**
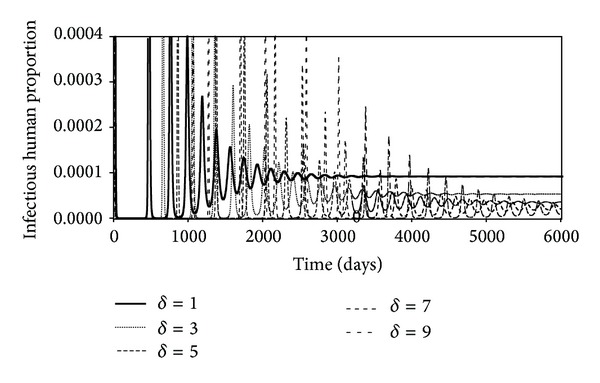
Time evolutions of the infectious human population for different increases in the death rate caused by the illness.

With a mathematical model to describe the progression of a disease, we can simulate the time course of an epidemic when the values of various parameters are different. This would allow us to gain insights into how the epidemic might respond to change in the practices which could change the values of the parameters. Recently, Aldila et al., [21] in the context of another disease (dengue fever), used mathematical modeling to determine the best strategy to control the spread of that disease. In the study, we look at the behaviors of the infectious humans when the percentage of contacts which are repetitive is changed, that is, *r*_*c*_. We have set *r*_*c*_ to 10%, 30%, 50%, 70%, and 90%. As *r*_*c*_ increases, the effective contact rate to create newly exposed susceptible decreases. This is clearly seen in Figure 7 where the maximum numbers of infectious humans (the peaks in each curve) decreases as *r*_*c*_ increases. The lower frame in Figure 7 is an expanded version of the top frame. It shows that increasing the value of *r*_*c*_ causes the equilibrium value of the infectious population to be lower. The equilibrium values are reached at an earlier time as *r*_*c*_ decreases (See bottom frame in Figure 7).  Figure 8 shows the time evolutions of the infectious populations when the death rate due to the virus increases. This happens when the virulence of the virus increases. Since the death rate of an infectious person is given as *d*_*h*_ + *δd*_*f*_, where *d*_*f*_ = 1/21, we have *δd*_*f*_ ≫ *d*_*n*_; death rates for the various curves are 1/21, 1/7, 5/21, 1/3, and 3/7 d^−1^. In Figure 8, we have plotted the simulated time evolution of the infectious humans when the fraction (*δ*) of infected humans placed into quarantine is increased. Exposed humans are not placed into quarantine since many of them will not become infectious and quarantine is a denial of a person's human rights. This denial cannot be justified on the basis of maybe he could become infectious. Looking at the time evolution of this population group, we see that the peak in the number and the number in the equilibrium state decrease as the fraction of infectious humans is increased.

*Sensitivity Analysis.* We apply the sensitivity analysis to show that the solutions are robust. We set all the parameters in the model to be the same but use different initial values of s*(0), e*(0), i*(0), q*(0), and r*(0). As we can see, all the trajectories (solutions) converge to the same epidemic equilibrium state (s*, e*, i*, q*, r*) = (0.0109, 0.00012, 0.000092, 0.000091, and 0.98). The results are as shown in Figure 10.

## 5. Conclusion

**Figure 9:**
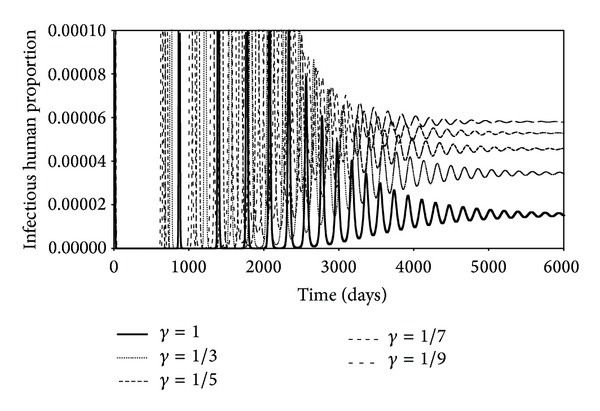
Time evolutions of the infectious human population for the different percentages of infectious humans being put into quarantine.

**Figure 10:**
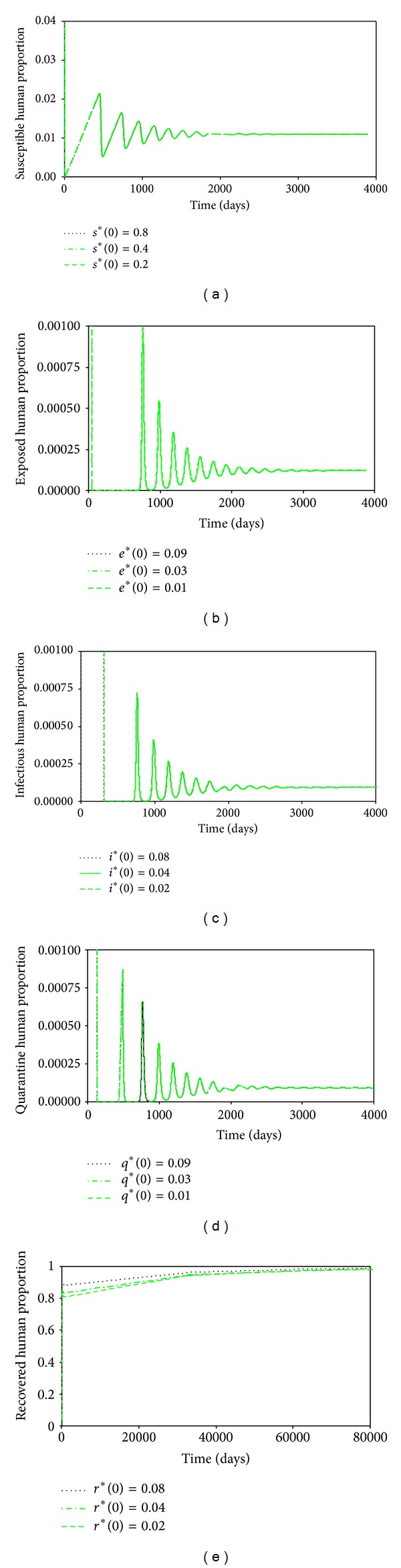
Trajectories of the different population groups when different initial values are used in the numerical simulation of the time behaviors of the population groups (sensitivity analysis).

Figures 7, 8, and 9 show the effects of changing the rate of repetitious contacts, the virulence of the virus, and the fraction of infectious humans placed into quarantine. Figure 7 shows that the number of infectious humans at equilibrium decreases as the rate repetition (*r*_*c*_) increases. Figures 8 and 9 indicate that the number of infectious humans at equilibrium decreases as the virulence or fraction of infectious humans increases. A further remark is that the repetition rate is difficult to control and that the increase or decrease of the virulence of the virus is beyond the control of public health officials. The only thing that can be controlled is the fraction of infectious humans that can be quarantined.

## Figures and Tables

**Table 1:** Definitions of our parameters in our dynamical equations.

Parameters	Definition
λ_h_	Birth rate of human population
r_c_	Percentage of contacts which are repetitive
*C*	Contact rate of H1N1 between the human population
*d*_*n*_	Natural mortality rate of human population
*δd*_*h*_	Increase in the mortality rate of human population caused by the disease per 1 time infection
ρ	Rate at which exposed human changes to become a recovered human
IP	Incubation period of H1N1 in human population
*K*	Rate at which quarantine human changes to become a recovered human
α	Rate at which infectious human changes to become a recovered human
γ	Rate at which infectious human changes to become a quarantine human
*N*(*t*)	Total human population at time *t*
